# Therapeutically harnessing cancer stem cell-derived exosomes

**DOI:** 10.18632/oncotarget.28542

**Published:** 2023-12-20

**Authors:** Yong Teng

**Keywords:** cancer stem cells; exosomes, the tumor microenvironment, therapeutic target, anticancer strategy

Cancer stem cells (CSCs), a small population of cancer cells capable of self-renewal, are thought to serve as a central hub for tumor initiation, growth, metastasis, and recurrence [1]. The potential for using CSCs in the diagnosis and treatment of cancer is gaining recognition. Exosomes are formed when multivesicular endosomes or multivesicular bodies fuse with the outer membrane of the cell, releasing various components such as DNA, RNA, lipids, metabolites, and cytosolic and cell surface proteins [2]. Over the past decade, our understanding of the characteristics and function of cancer-associated exosomes has expanded rapidly. As the major messengers, exosomes present in the tumor microenvironment (TME) play a critical role in maintaining the delicate balance between CSCs and non-CSCs [3]. Given the importance of CSCs, it is reasonable to believe that CSC-derived exosomes (CSC-Exos) are essential for communication between CSCs and other cells in the TME. Accumulating evidence has demonstrated that CSC-Exos contribute significantly to almost all fundamental aspects of cancer, including maintaining a continuous cycle of self-renewal within the TME, exerting control over neighboring or distant cells, enabling cancer cells to evade immune surveillance, and promoting immune tolerance [4].

Research indicates that CSC-Exos transport stemness-related factors, including OCT-4, SOX-2, NANOG, or lncRNA/microRNA, which in turn interact with neighboring cells to enhance stemness expression [5, 6]. Moreover, CSC-Exos have the ability to maintain the crosstalk between CSCs and non-CSCs, which may involve the transport of TGF-β [7]. In addition, CSC-Exos play a role in promoting neovascularization and metastasis. A study has shown that CD103^+^ cells direct CSC-Exos to clear cell renal cell carcinoma cells, and these exosomes carry miR-19b-3p into cancer cells, thereby inducing epithelial-mesenchymal transition (EMT) and facilitating metastasis [8]. Immune cells are vigilant in detecting and eliminating cells that undergo malignant changes. However, some of the transformed cells can evade immune surveillance and eventually form a tumor. Specifically, CSC-Exos enable cancer cells to evade immune surveillance and enable intercellular signaling within the immunosuppressive TME. On the other hand, CSC-Exos play an importance in promoting chemoresistance by delivering miRNAs, proteins and lipids to cancer cells, activating signaling pathways related to cell survival and growth, inducing EMT and remodeling the TME.

A deeper understanding of the characteristics and functions of CSC-Exos has the potential to lay the foundation for the development of novel clinical tools for diagnosis and prognosis, as well as therapies aimed at preventing tumor progression and recurrence. Recently, we have summarized research advances, explored opportunities and challenges, and outlined perspectives in this emerging field [4]. This work is intended to stimulate the exploration of innovative approaches, including the development of novel pharmaceuticals or interventions, to specifically target CSC-Exos and reduce their impact on cancer initiation and progression. While technical and regulatory challenges remain, recent significant advances in understanding the physiological and pathological functions of CSC-Exos have revealed numerous potential applications for diagnosis and treatment. These developments have accelerated the recognition of their broad clinical potential.
